# Compositions and antimicrobial properties of binary ZnO–CuO nanocomposites encapsulated calcium and carbon from *Calotropis gigantea* targeted for skin pathogens

**DOI:** 10.1038/s41598-020-79547-w

**Published:** 2021-01-08

**Authors:** G Ambarasan Govindasamy, Rabiatul Basria S. M. N. Mydin, Srimala Sreekantan, Nor Hazliana Harun

**Affiliations:** 1 Oncological and Radiological Sciences Cluster, Advanced Medical and Dental Institute, Universiti Sains Malaysia, Bertam, 13200 Kepala Batas, Pulau Pinang Malaysia; 2 Ann Joo Integrated Steel Sdn Bhd, Lot 1236, Prai Industrial Estate, 13600 Prai, Penang Malaysia; 3 Department of Biological Sciences, NUS Environmental Research Institute, National University of Singapore, 14 Science Drive 4, Singapore, 117543 Singapore; 4 School of Materials and Mineral Resources Engineering, Universiti Sains Malaysia, Engineering Campus, 14300 Nibong Tebal, Pulau Pinang Malaysia

**Keywords:** Microbiology, Diseases, Medical research, Materials science

## Abstract

*Calotropis gigantea (C. gigantea)* extract with an ecofriendly nanotechnology approach could provide promising antimicrobial activity against skin pathogens. This study investigates the antimicrobial capability of green synthesized binary ZnO–CuO nanocomposites from *C. gigantea* against non-MDR (*Staphylococcus aureus* and *Escherichia coli*) and MDR (*Klebsiella pneumoniae*, *Pseudomonas aeruginosa* and methicillin-resistant *S. aureus)* skin pathogens. Scanning electron microscopy and transmission electron microscopy revealed the size and shape of B3Z1C sample. Results of X-ray powder diffraction, energy-dispersive spectroscopy, FTIR and UV–Vis spectroscopy analyses confirmed the presence of mixed nanoparticles (i.e., zinc oxide, copper oxide, carbon and calcium) and the stabilising phytochemical agents of plant (i.e., phenol and carbonyl). Antimicrobial results showed that carbon and calcium decorated binary ZnO–CuO nanocomposites with compositions of 75 wt% of ZnO and 25 wt% CuO (B3Z1C) was a strong bactericidal agent with the MBC/MIC ratio of ≤ 4 and ≤ 2 for non-MDR and MDR pathogens, respectively. A significant non-MDR zone of inhibitions were observed for BZC by Kirby–Bauer disc-diffusion test. Further time-kill observation revealed significant fourfold reduction in non-MDR pathogen viable count after 12 h study period. Further molecular studies are needed to explain the biocidal mechanism underlying B3Z1C potential.

## Introduction

Ulcerative skin infections arising from the colonisation and development of Gram-positive bacteria, Gram-negative bacteria, and multidrug-resistant bacteria are significant health-care problems that seriously affect human skin. A prospective quantitative study reported that the prevalence rates of skin pressure ulcers (PUs) are 15.5% in Kuala Lumpur, Malaysia (2013)^1^, 33% in Palestine (2017)^2^, and 16% in Bandung, Indonesia (2017)^3^. Skin infection has been found in 60 (74.0%) of the collected samples from PUs of hospitalised patients, and these PUs primarily comprise *Enterobacteriaceae* strains (49.0%), such as *Escherichia coli* (*E*. *coli*), *Klebsiella pneumoniae* (*K*. *pneumoniae*), *Enterobacter* spp., and *Proteus* spp.; followed by *Staphylococcus aureus* (*S*. *aureus*) (28.0%) and nonfermenting GNB (23.0%), mostly *Pseudomonas aeruginosa* (*P*. *aeruginosa*), *Acinetobacter* spp., and methicillin-resistant *S. aureus* (MRSA)^4–7^. PUs are open infected wounds that develop on the skin as result of pressure on one spot of the body for too long or from friction on the skin. Some studies have found that new inorganic oxide antimicrobial agents synthesised from natural plants can be remarkable alternatives for infectious skin treatments of PUs because they are rich in numerous varieties of metal oxides that release ions and in reactive oxygen species (ROS), such as hydroxyl radical (·OH^−^) and superoxide (·O^2−^) which cause increased cell permeability, rupture, and death in microorganisms^8,9^.

The incorporation of inorganic metal and metal oxides in sponges^10^, hydrogels^11,12^, and bandages^13,14^ has become a research hotspot because of these materials’ advantages as antimicrobial agents for treating locally infected skin ulcers. Mixed inorganic metal and metal oxides are effective disinfectants because of their relatively nontoxicity, chemical stability, and efficient antibacterial activity (Table 1). The use of binary antimicrobial agents (e.g., CuO, ZnO, and Ag–ZnO) has been highlighted over single antimicrobial agents given the stronger synergic effect of the former in eliminating bacterial colonies at low concentrations^10,25,39^, more pronounced wound-healing ability^10^, lower cytotoxicity^10^, better biocompatibility^25^, and improved cell viability which indicates safe human application ^25^. The combined use of binary antimicrobial agents could reduce the cytotoxicity but not the antimicrobial effect^10,25^. Furthermore, several studies have shown that the incorporation of antimicrobial agents such as CuO^40^, CuSO_4_^41^, ZnO^42^, ZnO-SiO_2_^43^, and Re-ZnO^44^ into biopolymers can effectively combat Gram-positive and Gram-negative bacteria in a concentration-dependent manner. However, binary ZnO/CuO nanocomposites prepared from *Calotropis gigantea* (*C*. *gigantea*) leaves in the current work were found to exert a strong antimicrobial effect on multi-drug resistant (MDR) pathogens such as *P*. *aeruginosa* and MRSA compared with other previously reported antimicrobial binary inorganic oxides nanocomposites (Table 2). It can effectively work against MDR pathogens at a very low minimum bactericidal concentration (MBC) of about 0.3125 mg/mL.

**Table 1:** Antimicrobial properties of different mixed metal/metal oxides towards various microorganisms.

Mixed oxides	Route of synthesis	Size (nm)	Shape	Calcination temperature	Killing mechanism	Antimicrobial activity	Efficacy	Application	Toxicity	Refs
ZnO/CuO	Green route-*Theobroma cacao* seed bark extract	20–50	Spherical and rice grains	400 °C	Nil	Nil	Nil	Photocatalyst	Nil	^15^
CuO-ZnO	Biological route-*Cnicibenedicti*	28	Spherical	Nil	Nil	*S. aureus, E. coli, P. aeruginosa* and *C. albicans*	MIC: 0.3125–2.5%, MBC: 1.25–5%	Cidal	Nil	^16^
Cu-doped ZnO	Solution combustion-*Clerodendruminfortunatum* extract	17.49	Rod	200 °C	Generation of reactive oxygen species	*S. aureus, B. subtilis, Klebsiella and E. coli*	MIC: 0.04–0.95 mg/mL, ZOI: 8–17 mm	Therapeutic	Nil	^17^
Cu-doped ZnO	Solution combustion-*Clerodendruminerme*	20.73	Rod	200 °C	Generation of reactive oxygen species	*S. aureus, B. subtilis, Klebsiella and E. coli*	MIC: 0.03–0.09 mg/mL, ZOI: 13–20 mm	Therapeutic	Nil	^17^
ZnO/CuO	Green route-*Mentha longifolia* leaf extract	At 10 wt% CuO: 10, ZnO: 14	Spherical	60 °C	Nil	*S. aureus* and *E. coli*	*E. coli:* 10.16 mm, *S. aureus:* 17.1 mm at 10 wt% of ZnO/CuO	Antibacterial agents	Nil	^18^
CuO-ZnO	Sol–gel	15.99	Uniform particle	500 °C	Production of Zn^2+^ ions and reactive oxygen species	*S. aureus* and *E. coli*	*E. coli:* 2.3 mm, *S. aureus:* 2.1 mm at 25 wt% of ZnO/CuO	Antibacterial agents	Nil	^19^
Copper-doped ZnO	Depositions	50 and 100 and 100 and 600	Globular structure consisting of semicircular domes	Nil	Oxidative stress caused by ROS, Zn^2+^, Cu^0^, Cu^1+^, and Cu^2+^ ions released	*E. coli*	*E*. *coli* reduction below detection limits within 6 h under white light	Antibacterial agents	Nil	^20^
Zeolite\ZnO-CuO	Facile method	ZnO: 25.9, CuO: 56.2	CuO and ZnO formed on surface of zeolite cubic structure	450 °C	Release of Cu^2+^ and Zn^+2^ ions	*B. subtilis* and *E. coli*	*B*. *subtilis*: 18.9 mm and *E*. *coli*: 23.8 mm at 10 mg/mL under normal light at 24 h incubation	Antibacterial agents	Nil	^21^
Mesoporous silica SBA/CuZnO	Impregnation	2 µm	2D hexagonal and honeycomb structure	550 °C	Release of dissociated metal ions and the release of reactive oxygen species	*E. coli* and *S. aureus*	*E.* coli: MIC: 25 mg/mL and MBC: 100 mg/mL, *S. aureus*: MIC: 6.25 mg/mL and MBC: 50	Antibacterial agents	Nil	^22^
CS/Zn-Cu	Physico-chemical	1.7–23.7	Nil	60 °C	Nil	*B. cinerea*	ZOI: 1.7 cm at 90 μg/mL	Fungicidal	Nil	^23^
ZnO–CuO	Green route-*Calotropis gigantea* leaf extract	10–40	Spherical and hexagonal	500 °C	ROS	*S. aureus* and *E. coli*	ZOI: *S. aureus*: 6.74 mm and *E. coli*: 6.74 mm at 500 μg/mL; *S. aureus*: 8.25 mm and *E. coli*: 9.14 mm at 1000 μg/mL	Waste water treatment and biomedical	Nil	^24^
ZnO/Ag	Green route-*Mirabilis jalapa* leaf extract	19.3–67.4	Plates, sheets, and spherical	Nil	Activation of electrons; ions release and particle penetration	*P. aeruginosa, K. pneumoniae, E. coli, S. aureus and B. subtilis*	ZOI: *P. aeruginosa*: 18 mm*, K. pneumoniae*: 25 mm*, E. coli*: 17 mm*, S. aureus*: 20 mm *and B. subtilis*: 21 mm at 0.5 wt% of ZnO and Ag	Biomedical	Nil	^8^
Zinc oxide/silver- PVP/PCL	Oxalate decomposition	ZnO: 40.07 ± 9.70, Ag: 37.46 ± 12.02	Spherical	ZnO: 500 °C, Ag: 40 °C	Ag^+^ ions release and ZnO produces ROS	*S. aureus* and *E. coli*	Larger ZOI than single antibacterial agents	Antibacterial agents	No cytotoxicity against the human skin fibroblasts (HSFs), cell viability at 2 and 4 days: 90–95%	^25^
Ag/ZnO-CS	Deposition–precipitation	Length: 100–400 and width: 50–200	Rod-like structures	60 °C	Nil	*E. coli, S. aureus*, *P. aeruginosa*, DREC and MRSA	ZOI were captured	Wound dressing	CS-Ag/ZnO-0.5 tested against L02 cells, cell viability at 72 h incubation: 94%	^10^
Ag-ZnO	Green route- *Azadirachta indica* gum	15, pore diameter: 70–500	Spherical, porous and honeycomb structure	500 °C	Nil	Nil	Nil	Degradation of MB dye, green emitting LED	Nil	^26^
Ag/ZnO- cellulose fillers	Stepwise microwave assisted hydrothermal synthesis	ZnO: 1 µm; Ag: 100	ZnO: hollow and resemble hexagonal nuts; Ag: globular	40 °C	Silver ion and generation of reactive oxygen species	*E. coli* and *S. aureus*	Log CFU: *E. coli*: 6.2–> 7.1 and *S. aureus*: 4.2–> 5.1	Sanitary, hygienic or other interior	Nil	^27^
Ag-ZnO Bent-CS	Microwave-assisted synthesis	Ag: 9–30 and ZnO: 15–70	Aggregated particle	70 °C	Nil	*E. coli* and *E. faecalis*	Complete inactivation within first 2 min for ZnO (21 wt%) and Ag (3 .9 wt%)	Water disinfection	Nil	^28^
Honeycomb doped silver and zinc	Wet ceramic powder process in combination with co-firing	Nil	Honeycomb structure with a porous surface	Nil	Nil	*E. coli*	98.9–99.5% rates for Zn: 5–6% and Ag: 0.3%	Antibacterial agents	Nil	^29^
Ag–Cu	Green route-flower aqueous extract of *A. haussknechtii*	24.82 ± 4.85	Berries like	Nil	Electrostatic interaction and production of reactive oxygen species	*E. coli, S. aureus* and *P. aeruginosa*	ZOI: *E. coli*: 12.33 mm, *S. aureus*: 15 mm and *P. aeruginosa*: 15.33 mm; MIC: 5–25 μg/mL, MBC: 15–50 μg/mL	Antibacterial agents	Nil	^9^
Ag–Cu	Nanocasting	Core diameter: 25, Cu shell: 3.7	Rough pores	80 °C	Silver ions generate ROS and copper induces hydroxyl radicals	*E. coli* and *B. subtilis*	EC50: *E. coli*: 22.87 and *B. subtilis*: 23.33 after 24 h of incubation	Catalysis and antibacterial	Nil	^30^
Ag/Cu	Chemical reduction and impregnation	1–30 and 100–200	Spherical	200 °C	Penetration of Ag NPs, Ag^+^ and Cu^2+^ ions release	*C. albicans, E. coli* and *S. aureus*	99.99% and 100% after 5 and 15 washing/impregnation cycles	Bed linen and work wear	Nil	^31^
Ag–Cu/TNTs	Microwave assisted alkaline hydrothermal process and UV-photodeposition	TNTs: 7.5–10 thickness and ~ 5 inner diameter	Bundle	80 °C	Reactive oxygen species and superoxide radical anion	*S. aureus*	ZOI: 1Ag/1Cu TNT: 11.60 mm at 10 mg/mL and 25.40 mm at 20 mg/mL; complete inactivation within 90 min in visible light	Photocatalyst antibacterial agents	Nil	^32^
Cu-Ag	Green route-flower aqueous extract of *A. haussknechtii*	33.79 ± 18.73	Needle	Nil	Electrostatic interaction and production of reactive oxygen species	*E. coli, S. aureus* and *P. aeruginosa*	ZOI: *E. coli*: 12 mm, *S. aureus*: 15.33 mm and *P. aeruginosa*: 19 mm; MIC: 3–25 μg/mL, MBC: 5–50 μg/mL	Antibacterial agents	Nil	^9^
Ag-TiO_2_	Green route-flower aqueous extract of *A. haussknechtii*	36.99 ± 12.03	Spherical	Nil	Electrostatic interaction and production of reactive oxygen species	*E. coli, S. aureus* and *P. aeruginosa*	ZOI: *E. coli*: 12.5 mm, *S. aureus*: 16 mm and *P. aeruginosa*: 21.66 mm; MIC: 3–7 μg/mL, MBC: 5–25 μg/mL	Antibacterial agents	Nil	^9^
TiO_2_-Ag	Green route-flower aqueous extract of *A. haussknechtii*	35.55 ± 9.88	Cubic	Nil	Electrostatic interaction and production of reactive oxygen species	*E. coli, S. aureus* and *P. aeruginosa*	ZOI: *E. coli*: 12.66 mm, *S. aureus*: 15.66 mm and *P. aeruginosa*: 21 mm; MIC: 3–13 μg/mL, MBC: 5–25 μg/mL	Antibacterial agents	Nil	^9^
TiO_2_/ZnO-4A zeolite	Hydrothermal method and ion exchange process	10–50	Equiaxed	500 °C	Production of ROS; Zn^2+^ release and particle’s penetration	*S. aureus, P.* *fluorescens, L. monocytogenes* and *E. coli*	MIC: 1–2 mg/mL, MBC: 2–3 mg/mL; ZOI: 9.22–10.73 mm	Packaging in food industry	Nil	^33^
ZnO/TiO_2_	Precipitation method and sol–gel	100	No defined shape	500 °C	Zn^2+^ ions release	*S.aureus,* *E. coli, K. pneumoniae, P. aeruginosa, S* *paratyphi A* and *C. albicans*	CFU reduction %: *S. paratyphi A*: 1.02–2.38, *E. coli*: 32.54–39.33, *K. pneumoniae*: 87.88– 92.04*, P. aeruginosa*: 33.21–42.26, *S.aureus*: 100 and *C. albicans*: 28.16–50	***Antibacterial gents***	Nil	^34^
Au-CuO	Biological synthesis using using Cnicibenedicti	13	Spherical	Nil	Nil	*S. aureus, E. coli, P. aeruginosa* and *C. albicans*	MIC: 1.25–2.5%, MBC: 2.5%	Cidal	Nil	^16^
Graphene-ZnO	Green route-*Crocus sativus* petal extract	25	Spherical	100 °C	Ion release and production of reactive oxygen species	*S. aureus* and *E. coli*	MIC: *S. aureus*: 62.5 μg/mL and *E. coli*: 125 μg/mL; MBC: *S. aureus*: 125 μg/mL and *E. coli*: 500 μg/mL	Antioxidant and antibacterial in the pharmacy	Nil	^35^
Cu/Pd	Facile method	3	Hexagonal	Nil	Metal ions release	*E. coli*, *P. aeruginosa*, *E. faecalis* and *S. aureus*	ZOI: 9.16– 15.91 mm; MIC: 46.98–375.9 μg/mL	Biomedical and industrial	Nil	^36^
Ag/Fe	Green route-palm dates fruit	5–40	Irregular-truncated triangular polyhedral nano-disks and spherical	50 °C	Electrostatic interaction of ions	*S. aureus* and *E. coli*	MIC: *S. aureus*: 60 μg/mL and *E. coli*: 80 μg/mL; ZOI: *S. aureus*: 25 mm and *E. coli*: 20 mm at 20 μg/mL	Antibacterial agents	Nil	^37^
Zinc oxide/gentamicin-CS	Forced hydrolysis and coating	15	Polyhedral	80 °C	Nil	*S. aureus* and *P. aeruginosa*	ZOI: *S. aureus*: 17 mm and *P. aeruginosa*: 17 mm; MIC: *S. aureus*: 0.12 μg/mL and *P. aeruginosa*: 0.97 μg/mL	Photodiagnosis or biosensing	Nil	^38^

**Table 2:** MIC and MBC concentration of binary antimicrobial agent.

Binary antimicrobial agent	Particle size (nm)	Type of strain	MIC (mg/mL)	MBC (mg/mL)	Refs.
ZnO/CuO	30	*P*. *aeruginosa*	2.048	> 4.096	^45^
		*E*. *coli*	2.048	> 4.096	
		*S*. *aureus*	2.048	> 4.096	
CuO-ZnO	28	*P*. *aeruginosa*	2.5	5	^16^
		*E*. *coli*	0.625	1.25	
		*S. aureus*	0.3125	1.25	
Au-CuO	13	*P*. *aeruginosa*	2.5	2.5	^16^
		*E*. *coli*	1.25	2.5	
		*S. aureus*	2.5	2.5	
TiO_2_/ZnO/4A	10–50	*E. coli*	1	2	^33^
		*S. aureus*	2	3	
TiO_2_/ZnO	–	*E*. *coli*	5	10	^46^
		*S. aureus*	5	10	
		*K. pneumoniae*	5	10	
		*MRSA*	0.15	0.30	
ZnO-CuO	Length: 8.126 and diameter: 7.515	*P*. *aeruginosa*	0.15625	0.3125	“This work”
		*E. coli*	0.625	2.5	
		*S*. *aureus*	0.625	2.5	
		*K. pneumoniae*	0.625	1.25	
		MRSA	0.15625	0.3125	

Accordingly, the present study focused on the preparation of green synthesised binary ZnO-CuO nanocomposites using *C. gigantea* leaf extract. The microbial activity of these nanocomposites was investigated by culturing with skin ulcer pathogens such as *E. coli*, *K. pneumoniae*, *S. aureus*, *P. aeruginosa*, and MRSA. Furthermore, the effects of different compositions on ZnO-CuO nanocomposites were explored with respect to their prospective antimicrobial application.

## Materials and methods

### Preparation of leaf extract and binary inorganic oxides

Whole *C. gigantea* plant was collected from Perai Pulau Pinang, Malaysia and identified by an expert from the Unit Herbarium, Pusat Pengajian Sains Kajihayat USM Pulau Pinang (Herbarium No.: 11843). *C. gigantea* leaves were extracted using deionised water and boiled using hot plate^47,48^. Then, the filtered leaf extracts were taken and boiled with a stirrer–heater. Binary ZnO–CuO nanocomposites were prepared by adding copper (II) nitrate trihydrate and zinc nitrate hexahydrate into the extract solutions simultaneously and then boiled until they were reduced to pastes. These pastes were calcined in an air-heated furnace^47,48^. Notably, the mixing composition of copper (II) nitrate trihydrate and zinc nitrate hexahydrate was varied with constant rotation speed and calcination temperatures (Table 3). The samples prepared at weight percentages of 25 wt%, 50 wt%, and 75 wt% of zinc nitrate hexahydrate were denoted as B1Z3C, B1Z1C, and B3Z1C, respectively. Commercial B3Z1C sample was prepared by mixing ZnO (< 100 nm; Aldrich) and CuO (< 10 µm; Sigma–Aldrich) with an agate mortar (Table 3).

**Table 3:** Composition of binary ZnO-CuO nanocomposites samples.

No	BZC sample	ZnO (g)	CuO (g)
1	B1Z3C	1.25	3.75
2	B1Z1C	2.5	2.5
3	B3Z1C	3.75	1.25
4	B3Z1C (commercial)	3.75	1.25

### Physicochemical characterisation

The crystal phases of BZC nanocomposites were studied by X-ray diffraction (XRD; Bruker D8 powder diffractometer) operated in reflection mode with a Cu Kα radiation (40 kV, 30 mA) diffracted beam monochromator. The step scan mode with a step size of 0.030° within the range of 10° to 90° was used. Scanning electron microscopy (SEM; Fei Quanta FEG 650) was used for morphology and microstructure observations of BZC nanocomposites. The purity of BZC was identified by energy-dispersive X-ray (EDAX) spectroscopy which was equipped with SEM. Detailed morphology of B3Z1C nanocomposites was further confirmed by transmission electron microscopy (TEM; FEI TECHNAI F20 G2).The characteristic optical properties of BZC nanocomposites were studied using a UV–Vis spectrophotometer (Varian) at room temperature within the range of 200–900 nm. FTIR spectroscopy (Perkin Elmer) was recorded within the range of 4000–400 cm^−1^ through the KBr pellet method to observe the functional groups involved in the natural-plant green synthesis and stabilization of B3Z1C nanocomposites.

### Minimum inhibitory concentration (MIC)/MBC determination and tolerance level

Antibacterial activity of BZC nanocomposites against *S. aureus* 29213, *E. coli* 25922, *P. aeruginosa* 27853, *K. pneumoniae* 700603, and MRSA 38591 were assessed using broth-dilution method on 96-well plates as described by Harun et al^46^. Absorbance was read at 980 nm wavelength^46^. High wavelength was selected because of BZC nanoparticle deposition. The bactericidal and bacteriostatic capacity of the samples was determined by the tolerance level^46^.

### Time-kill assay

The antibacterial activity of BZC nanocomposites against time was performed using time-kill assay as illustrated in a previous protocol^46^. *S. aureus* bacterial suspension adjusted to 0.5 McFarland standard turbidity was used and diluted with sample solution to a final concentration of 2.5 mg/mL.

### Kirby–Bauer disc-diffusion test

The antibacterial activity of BZC nanocomposites against *S*. *aureus* was further evaluated using Kirby–Bauer disc-diffusion test^49^. BZC nanocomposite solutions (2.5 and 10 mg/mL) were prepared and used further for antibacterial studies. About 20 µL of BZC nanocomposite solution, negative control (10% DMSO + distilled water), and *C*. *gigantea* leaf extract were loaded into 6 mm sterile filter papers, and the solution was allowed to be diffused within 15–30 min. Then, all discs were properly placed on agar which was already previously spread with bacterial culture. A standard antibiotic comprising 10 µg of Oxoid streptomycin antimicrobial susceptibility discs served as a positive control. After 24 h of incubation at 37 °C, the different levels of zone of inhibition were measured.

## Results and discussion

### Surface morphology of binary ZnO–CuO nanocomposites

**Figure 1:**
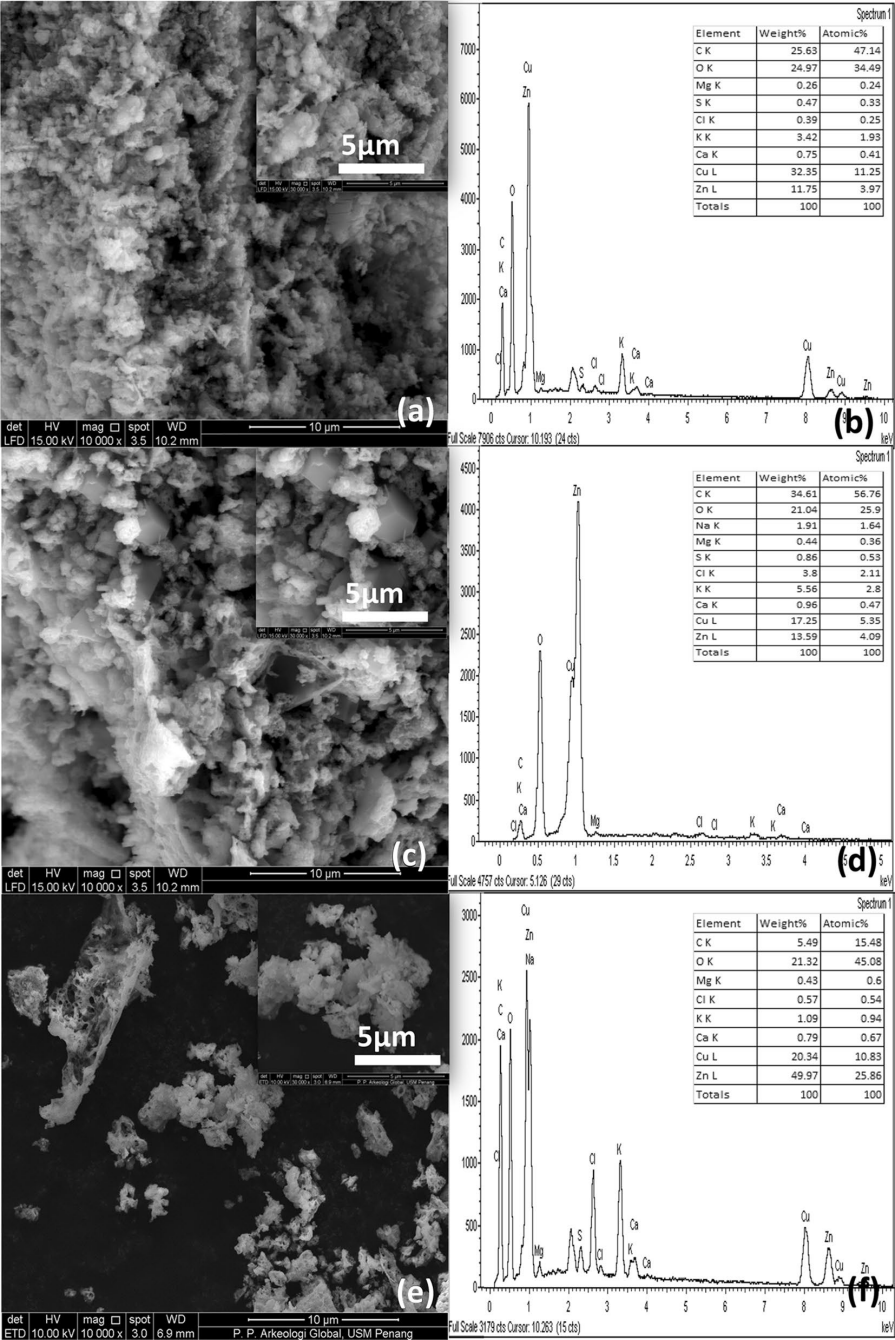

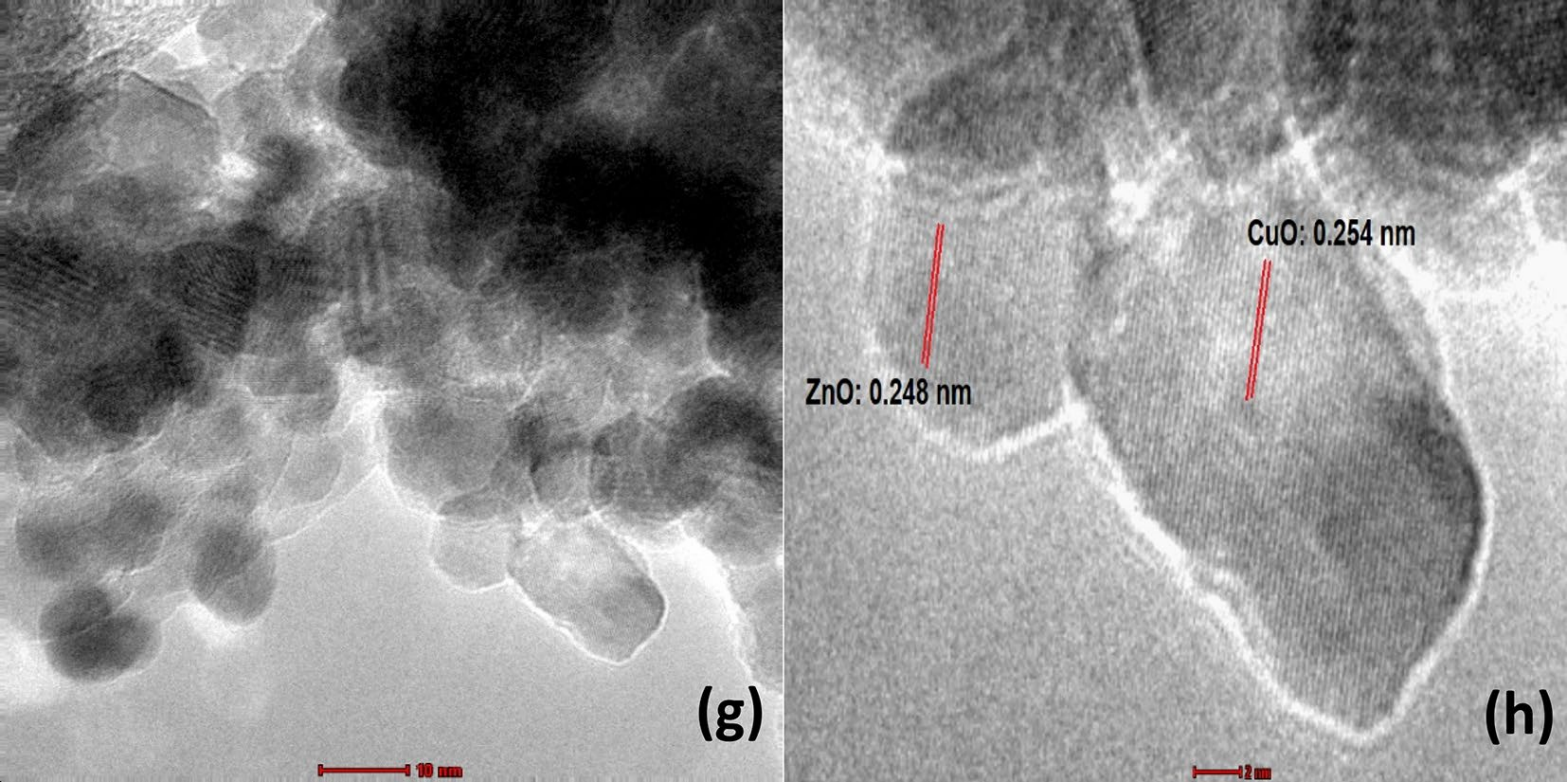
Morphology of BZC nanocomposites; (**a**) SEM image of B1Z3C (10.00 µm), (**b**) EDAX of B1Z3C, (**c**) SEM image of B1Z1C (10.00 µm), (**d**) EDAX of B1Z1C, (**e**) SEM image of B3Z1C (10.00 µm), (**f**) EDAX of B3Z1C, (**g**) TEM image of B3Z1C (10 nm) and (**h**) Magnified TEM image of B3Z1C nanocomposites along with lattice fringes (2 nm).

The SEM images of BZC nanocomposites are shown in Fig. 1. B1Z1C had a porous nature (Fig. 1c) with few irregular rod-shaped particles (inset in Fig. 1c). Meanwhile, B1Z3C (Fig. 1a) and B3Z1C (Fig. 1e) had porous honeycomb structures with agglomerated morphology (inset in Fig. 1a,e). The large porous honeycomb structures further increased the available surface area for antimicrobial activity^26^. These uniform pores were produced during green synthesis owing to the escape of gases at high temperatures^26^. The EDAX profile of the green synthesised B3Z1C nanocomposites confirmed the presence of Zn, Cu, and O, which were about 49.97 wt%, 20.34 wt%, and 21.32 wt%, respectively. Some weak signals for C, Mg, S, Cl, K, Na, and Ca atoms were found for all BZC nanocomposites (Fig. 1b,d,f). Similar results have been reported for green nanoparticles derived from *Artemisia haussknechtii* leaf extract^50^, aqueous *Artemisia haussknechtii* flower extract^9^, *Protoparmeliopsis muralis lichen*^51^, *Ochradenus baccatus* leaves^52^, and *Jatropha curcas* L. leaf^53^. The presence of elements such as C, Mg, S, Cl, K, Na, and Ca in small amounts indicated the participation of plant phytochemical groups in reducing and capping the green synthesised BZC nanocomposites^9,50–53^. Meanwhile, the TEM image of B3Z1C nanocomposites revealed irregular oval and quasi-spherical shape with an average length of 8.126 nm and diameter of 7.515 nm in size (Fig. 1g). These structures could increase the available surface area for reaction. The magnified TEM image of the B3Z1C nanocomposites along with the lattice fringes with an interfringe distance of 0.248 and 0.254 nm belonged to ZnO and CuO, respectively (Fig. 1h).

### Crystal analysis of binary ZnO–CuO nanocomposites

**Figure 2:**
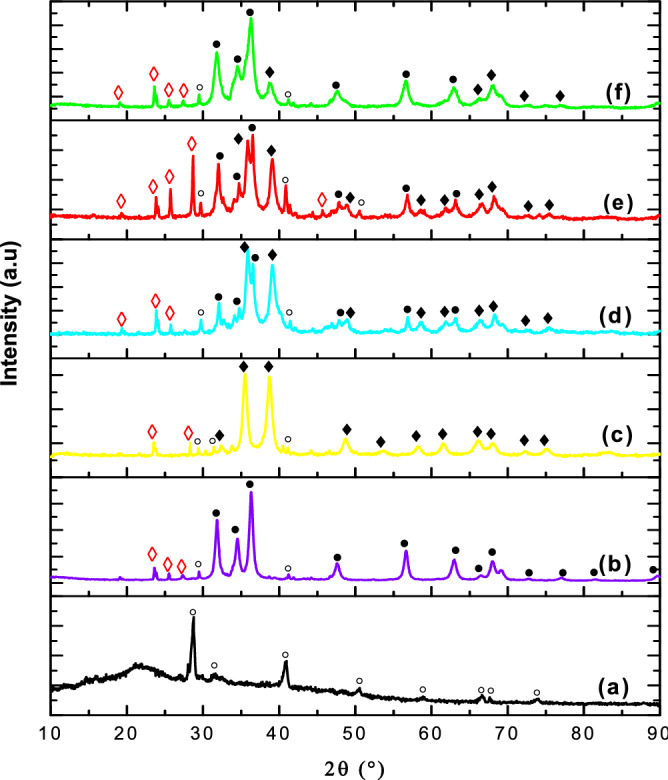
XRD diffraction peaks of BZC nanocomposites prepared at different composition. (**a**) *C*. *gigantea* leaves powder, (**b**) Green ZnO, (**c**) Green CuO, (**d**) B1Z3C, (**e**) B1Z1C and (**f**) B3Z1C [open circle: *C*. *gigantea* leaves, filled balck circle: ZnO, filled black rhombus: CuO, open red rhombus: additional peaks after green synthesis].

Prominent diffractive peaks on the differential ratio of binary ZnO–CuO nanocomposites were indexed by comparing the green ZnO and CuO diffraction angle 2θ with ICDD ZnO 01-089-0510 and ICDD CuO 01-089-5897, as presented in Fig. 2. Green CuO was observed to have 12 characteristic peaks at 32.32°, 35.50°, 38.71°, 45.01°, 48.37°, 53.29°, 58.15°, 61.09°, 65.56°, 67.90°, 72.16°, and 75.13°, which corresponded to the crystal surfaces (110), (− 111), (111), (202), (− 202), (020), (202), (− 113), (− 311), (220), (311), and (004), respectively. It had the following lattice parameters: a = 4.686486, b = 3.421156, c = 5.129263, α = 90°, β = 99.413°, γ = 90°, and d-spacing of 2.52761 Å with a monoclinic crystalline structure. Green ZnO was observed to have 12 characteristic peaks at 31.87°, 34.57°, 36.37°, 47.62°, 56.68°, 62.92°, 66.43°, 68.02°, 72.28°, 76.87°, 81.04°, and 89.44°, which corresponded to the crystal surfaces (100), (002), (101), (102), (110), (103), (200), (201), (004), (202), (104) and (203), respectively. It had the following lattice parameters: a = 3.252352, b = 3.252352, c = 5.209155, α = 90°, β = 90°, γ = 120°, and d-spacing of 2.47193 Å with a hexagonal wurtzite crystalline structure.

Meanwhile, six characteristic peaks of ZnO for sample B3Z1C were identified at 31.72°, 34.45°, 36.25°, 47.35°, 56.41°, and 62.71° and deemed to correspond to the (100), (002), (101), (102), (110), and (103) crystal surfaces, respectively. Two other characteristic peaks of CuO at 38.62° and 67.78° were found and deemed to correspond to the (111) and (220) crystal surfaces, respectively. For sample B1Z3C, the peaks at 31.72°, 34.45°, 36.25°, 47.35°, 56.41°, 62.71°, and 68.05° belonged to the (100), (002), (101), (102), (110), (103), and (201) indices of ZnO nanoparticles, respectively. The diffractive peaks of CuO detected at 35.68°, 38.62°, 58.33°, 61.27°, and 65.80° corresponded to the (–111), (111), (202), (–113), and (–311) crystal surfaces, respectively. All 2θ values of ZnO and CuO for BZC nanocomposites slightly shifted, indicating that some modifications of ZnO with CuO occurred and a strong crosslinking framework structure of Zn–O–Cu atoms formed. Moreover, the binary mixing of CuO and ZnO resulted in decreased crystallinity of BZC nanocomposites. The peak intensity drastically increased with increased amount of ZnO or CuO in the BZC nanocomposites (Fig. 2), thereby indicating the variation in composition (25 wt%, 50 wt%, and 75 wt% of ZnO) during green synthesis. A few additional peaks were observed at 23.65°, 25.69°, 27.73°, 29.47°, and 40.78° (Fig. 2). This finding was possibly due to the presence of the phytochemical element of *C. gigantea* leaves as a capping and reducing agent^47^. The XRD patterns of powdered *C*. *gigantea* leaves successfully revealed trace natural elements such as calcium and carbon (Fig. 2). *C*. *gigantea* natural plant is rich in calcium and carbon elements. Calcium was observed to have six characteristic peaks at 28.80°, 50.47°, 58.89°, 66.70°, 67.70°, and 73.92°. The additional peaks detected at 31.53° and 40.94° were attributed to the natural graphene-like carbon present in the BZC nanocomposites^54^ as carbon is the main phytochemical element in the leaves of the *C. gigantea* medicinal plant^55^.

The main novelty of this study was the detection of pythochemical elements such natural calcium^56^ and carbon^54,57^ in leaf extract, which could further boost the antimicrobial activity of BZC nanocomposites. Calcium and carbon elements have never been reported before in the studies of Sharma et al., Gawade et al., and C R Rajith Kumar et al. performed on the same *C*. *gigantea* medicinal plant^24,47,48^.

### FT-IR analysis of binary ZnO–CuO nanocomposites

**Figure 3:**
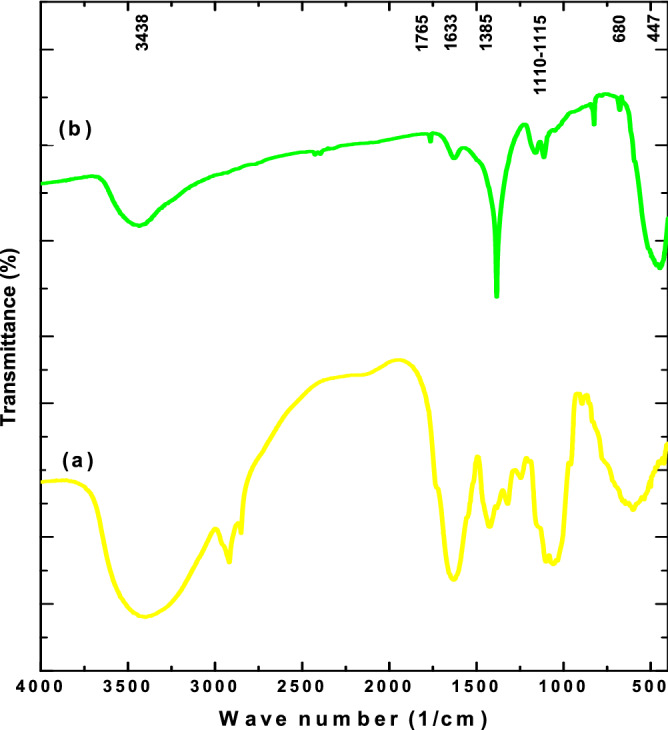
FTIR spectra (**a**) *C*. *gigantea* leaves and (**b**) B3Z1C nanocomposites.

The FTIR spectra of B3Z1C nanocomposites and *C*. *gigantea* leaves are shown in Fig. 3. The presence of capping and stabilization agents such as flavonoids, polyphenolics, and terpenoids can be confirmed from this analysis. The weak absorption band at 447 cm^–1^ was characteristic of the ZnO functional group^58,59^. However, the CuO functional group was not visible owing to its low composition in the B3Z1C nanocomposite binary system. The spectra further showed a very intense band at 3438 cm^−1^ associated with the O–H stretching polyphenols (flavonoids) present in the plant extract. The characteristic peaks at 1633 and 1765 cm^−1^ can be attributed to C=C (carbonyl group) and C=O stretching, respectively. The absorption band between 1110 and 1115 cm^−1^ could be attributed to C–O stretching owing to the biomolecules of *C*. *gigantea* leaves. The broad absorption band at 1385 cm^−1^ was observed owing to the O–C–O stretching modes of vibration of esters. The absorption band observed at 680 cm^−1^ belonged to primary amines, indicating proteins. Therefore, the presence of phenolic and carbonyl compounds of *C*. *gigantea* leaves played vital roles in the stabilisation of green B3Z1C nanocomposite formation and antimicrobial activity^15^.

### UV–Vis spectroscopy analysis of binary ZnO–CuO nanocomposites

**Figure 4:**
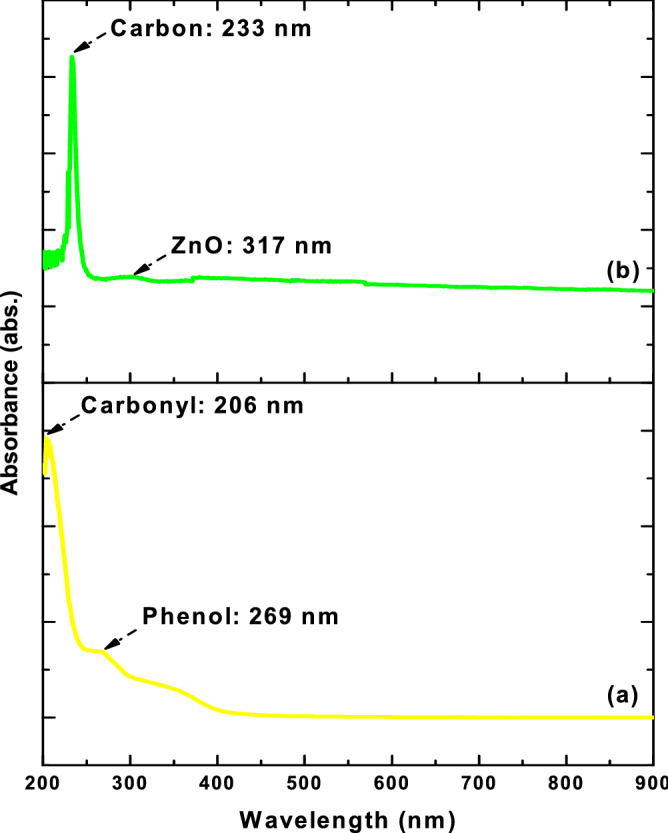
UV–Vis diffuse reflectance spectra (**a**) *C*. *gigantea* leaves and (**b**) B3Z1C nanocomposites.

The UV–Vis diffuse reflectance spectra of *C*. *gigantea* extract and B3Z1C nanocomposites are shown in Fig. 4. The appearance of a small broad peak at approximately 317 nm indicated the formation of irregular oval and quasi-spherical B3Z1C nanocomposites. Absorption peaks at 206 nm could be attributed to various chromophores, including the C=C bond of various compounds, the C=O bond of carbonyl compounds, and the benzene ring, whereas the absorption peak at 269 nm may be related to the various aromatic compounds, such as phenolics^60^. A sharp distinct peak was found at 233 nm owing to the formation of natural graphene-like carbon which played an important role in antimicrobial efficacy against MDR strains^61^.

### Antimicrobial properties of binary ZnO–CuO nanocomposites

About 37% of patients with skin-ulcer disease are infected with Gram-positive *S. aureus* pathogen^62^. The antimicrobial characterisation BZC nanocomposites with different ratios is presented in Fig. S1 and Table 4. The MICs of B1Z3C, B1Z1C, and B3Z1C were 5, 2.5, and 0.625 mg/mL for *S. aureus*, respectively. Similar to the MIC values, B1Z3C and B1Z1C had MBCs of 20 mg/mL, and the counterpart for B3Z1C was 2.5 mg/mL for *S. aureus*. B3Z1C exerted a higher bactericidal effect against the *S. aureus* strain at the lowest MIC/MBC values (0.625 mg/mL/2.5 mg/mL). Antimicrobial activity was further enhanced by increasing the amount of ZnO nanoparticles in the binary compound (ZnO-CuO). This finding can be explained by the fact that the binary B3Z1C nanocomposites were highly diffusible and able to generate more Zn^2+^ ions^19^. Moreover, Cu^2+^ ions bound the cell wall of host cells through surface proteins and entered the cell^19^. Subsequently, the change in cell metabolism led to the microbe’s cell death^19^. Commercial B3Z1C was also prepared and tested against *S*. *aureus* for comparison. Results showed that commercial B3Z1C was a bacteriostatic agent because the MBC/MIC ratio was ≥ 16^46^ (Table 4 and Fig. S1). However, the green B3Z1C was labelled as a strong bactericidal agent because the tolerance ratio was ≤ 4.

**Table 4:** MIC and MBC of BZC nanocomposites against *S. aureus*.

Samples	MIC (mg/mL)	MBC (mg/mL)	MBC/MIC
B1Z3C	5	20	4
B1Z1C	2.5	20	8
B3Z1C	0.625	2.5	4
B3Z1C (commercial)	0.625	10	16

Further antimicrobial analysis of B3Z1C nanocomposites was conducted on selected skin-ulcer pathogens, and results are shown in Table 5. These pathogens are commonly associated with skin-ulcer disease^4–7^. Also, the inhibitory activities of binary antimicrobial agents on bacterial colonies highly depend on the antimicrobial efficacy of dual-ionic systems and types of microbial pathogens, such as non-MDR Gram-positive bacteria (*S*. *aureus*), Gram-negative bacteria (*E. coli*) and MDR bacteria (*P. aeruginosa*, *K. pneumoniae*, and MRSA). The MIC amounts for B3Z1C were 0.625, 0.15625, 0.625, and 0.15625 mg/mL for *E. coli*, *P. aeruginosa*, *K. pneumoniae*, and MRSA, respectively. MBC values with 2.5, 0.3125, 1.25, and 0.3125 mg/mL were also observed for this green binary inorganic oxide sample. Table 5 indicates that for all tested microbes, the tolerance levels for B3Z1C were less than 4, indicating that the sample was a strong bactericidal agent. Binary B3Z1C has strong antimicrobial activity against Gram-negative bacteria (*E*. *coli*). Table 5 is the evidence for this finding. Clearly, B3Z1C showed very promising results against all tested MDR microbes such as *P. aeruginosa*, *K. pneumoniae*, and MRSA. This outcome may be due to the B3Z1C nanoparticles’ larger surface-to-volume ratio and the cell-membrane penetration of the bacteria by its ions. Some studies have reported that the antimicrobial effectiveness of green synthesised inorganic oxide nanoparticles depends on high particle dosage and small nanoparticle size, which could explain the higher antimicrobial activities of B3Z1C. The antimicrobial activity of B3Z1C was due to the electrostatic interaction between positively charged zinc and copper ions (Zn^2+^ and Cu^2+^) and negatively charged microbial cell membranes^21^. The antimicrobial activity of B3Z1C nanocomposites relied on the generation of ROS as well^17,19^. Moreover, free ions from natural organic carbon and calcium derived from *C*. *gigantea* leaf extract played an important role in exerting the synergic effect that killed MDR microbes at very low concentrations^54,56^.

**Table 5:** MIC and MBC of B3Z1C nanocomposites against different microbes.

Strain	MIC (mg/mL)	MBC (mg/mL)	MBC/MIC (mg/mL)
*S. aureus* 29213	0.625	2.5	4
*E. coli* 25922	0.625	2.5	4
*P. aeruginosa* 27853	0.15625	0.3125	2
*K. pneumoniae* 700603	0.625	1.25	2
MRSA 38591	0.15625	0.3125	2

**Figure 5:**
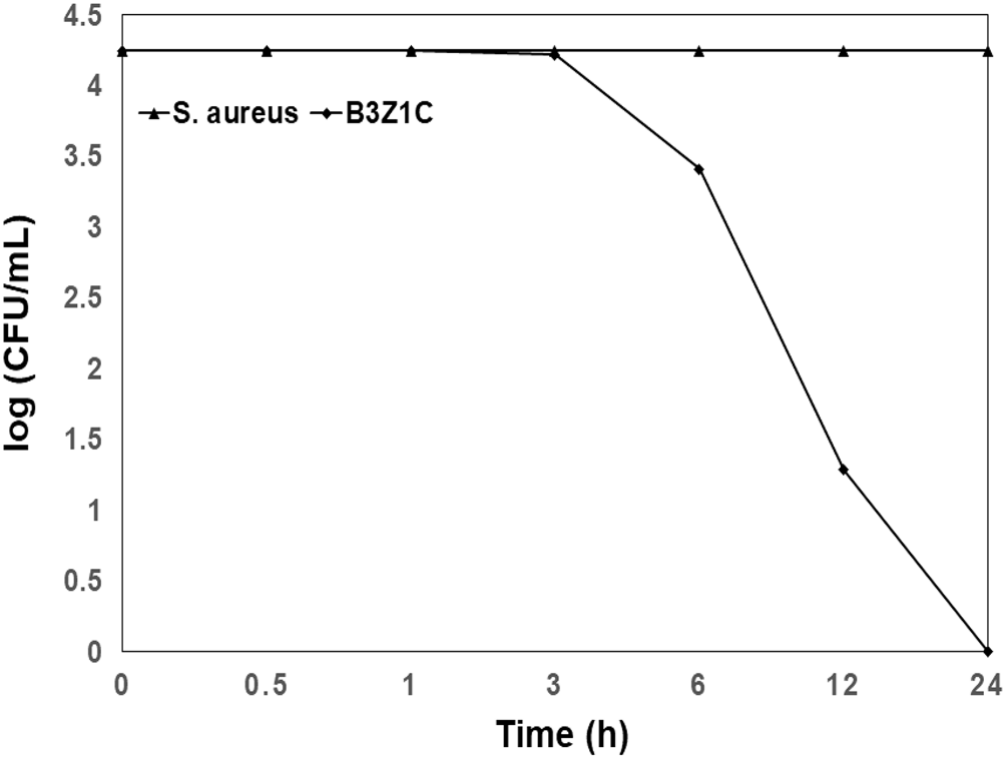
Time-kill curves against *S. aureus* strains using 2.5 mg/mL of green B3Z1C sample for 0.5 h (30 min), 3 h, 6 h, 12 h and 24 h treatment periods. These data represent mean (± SD) of three replicates.

Results of time-kill assay were presented in terms of the changes in log_10_ CFU/mL of viable *S. aureus* colonies, as shown in Fig. S2. The green synthesised B3Z1C nanocomposites were found to have significant bactericidal activity. Figure 5 presents the time-kill curve graph for the strain. Generally, bacterial growth includes a log or exponential phase in which bacterial-cell doubling occur and their biomass increases from day 1 to day 2^63,64^. A reduction in viable count from 4.3 log_10_ to 3.4 log_10_ was observed after 6 h of incubation for *S. aureus*. By 12 h, only 1.3 log_10_ of bacterial colonies were found. At 24 h, the bacteria were completely killed. Thus, Gram-positive *S. aureus* bacteria were effectively controlled by the synergistic combination of 75 wt% of ZnO and 25 wt% of CuO nanoparticles in the presence of natural graphene-like carbon, calcium, and phytochemical constituents such as cardiac glycosides, tannins, saponins, terpenes, flavonoids, and phenolics in *C. gigantea* leaf extract^54,56,65–68^.

Furthermore, Kirby–Bauer disc-diffusion method was used to evaluate the antimicrobial activity of BZC nanocomposites against Gram-positive *S*. *aureus*. The cultures exposed to negative control sample did not show any inhibition zones around the filters, indicating that they did not have any antibacterial properties. However, B3Z1C exhibited a wider zone of inhibition (ZOI) than other BZC samples possibly because of the nanoparticle size and the fast diffusion of metal ions into agar medium (Fig. S3 and Table 6). The antimicrobial activity of all green BZC samples further improved with increased concentration. *C*. *gigantea* extract also exhibited a slight ZOI toward *S*. *aureus* which could be attributed to bioactive compounds such as carbonyl and phenolic groups. The antibiotic streptomycin serving as a positive control exhibited a larger ZOI, as shown in Fig. S3 and Table 6.

**Table 6:** Kirby–Bauer disc diffusion ZOI (mm) of BZC nanocomposites against *S*. *aureus*.

Sample	ZOI (mm) at 2.5 mg/mL	ZOI (mm) at 10 mg/mL
Negative control	NA	NA
B1Z3C	6	6.67
B1Z1C	6.67	7
B3Z1C	6.67	7.33
B3Z1C (commercial)	6	7
*C*. *gigantea* extract	6.17	6.17
Streptomycin-10 µg	13	

These data represent mean (± SD) of three replicates. NA symbolises no bacterial activity found in this work.

## Conclusions

Binary B3Z1C nanocomposites prepared at compositions of 75 wt% of ZnO and 25 wt% CuO demonstrated significant antimicrobial property against non-MDR and MDR pathogens with tolerance ratio of ≤ 4 and ≤ 2, respectively. Besides, promising antimicrobial effect of B3Z1C sample towards non-MDR bacteria (*S*. *aureus*) were seen from disc diffusion assay and time kill analysis. The mechanisms underlying the biocidal activity of B3Z1C nanocomposites may involve the presence of natural carbon, free ions (i.e., Cu^2+^, Zn^2+^ and Ca^2+^), and ROS. Further In vitro* and *In vivo toxicity studies are needed to understand B3Z1C efficiency in treating PU infections.

## Supplementary Information

Supplementary Information.

## Data Availability

The datasets generated and/or analysed during the current study are not publicly available due to the patent application for methods of making and using and compositions of binary nanocomposites formed by green synthesis but are available from the corresponding author on reasonable request.
