# Supplementary material for: Compositions and antimicrobial properties of binary ZnO–CuO nanocomposites encapsulated calcium and carbon from Calotropis gigantea targeted for skin pathogens
Source: Sci Rep. 2021 Jan 8;11:99. doi: 10.1038/s41598-020-79547-w (PMC7794424; doi:10.1038/s41598-020-79547-w)
Supplement: Supplementary file 1 — Supplementary Information. [file 41598_2020_79547_MOESM1_ESM.docx]

**Compositions and antimicrobial properties of binary ZnO****–CuO nanocomposites encapsulated calcium and carbon from *Calotropis gigantea* targeted for skin pathogens**

**G Ambarasan Govindasamy^1,2^, Rabiatul Basria S. M. N. Mydin^1,3*^, Srimala Sreekantan^4*^ & Nor Hazliana Harun^1^**

^1^Oncological and Radiological Sciences Cluster, Advanced Medical and Dental Institute, Universiti Sains Malaysia, 13200 Bertam, Kepala Batas, Pulau Pinang, Malaysia.

^2^Ann Joo Integrated Steel Sdn Bhd, Lot 1236, Prai Industrial Estate, 13600 Prai, Penang, Malaysia.

^3^Department of Biological Sciences, National University of Singapore, 14 Science Drive 4, 117543 Singapore.

^4^School of Materials and Mineral Resources Engineering, Universiti Sains Malaysia, Engineering Campus, 14300, Nibong Tebal, Pulau Pinang, Malaysia.

*Correspondence: [rabiatulbasria@usm.my](mailto:rabiatulbasria@usm.my) and [srimala@usm.my](mailto:srimala@usm.my)

**Supporting Information**

| **Pathogenic bacteria** | **Negative**  **control** | **B1Z3C**  **5 mg/mL** | **B1Z3C**  **10 mg/mL** | **B3Z1C**  **2.5 mg/mL** | **B3Z1C**  **1.25 mg/mL** | **B3Z1C**  **0.625 mg/mL** | **B3Z1C (commercial)**  **2.5 mg/mL** |
| --- | --- | --- | --- | --- | --- | --- | --- |
| ***S. aureus*** | 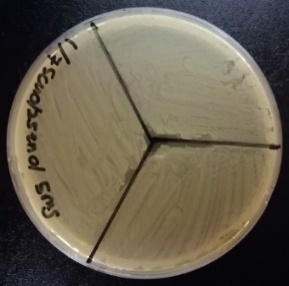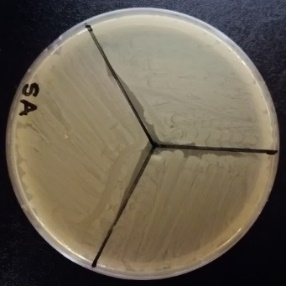  **(a)** | 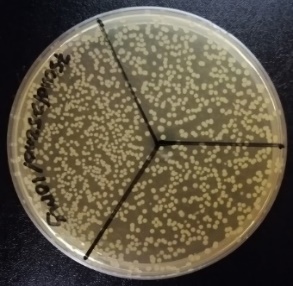  **(b)** | **(c)** | 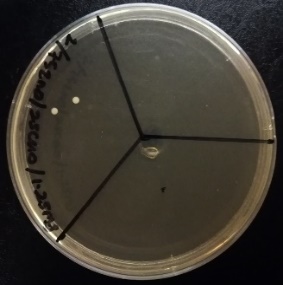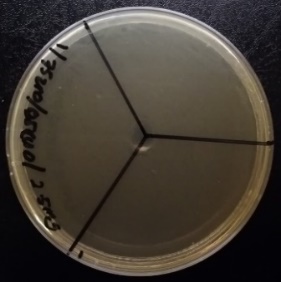  **(d)** | 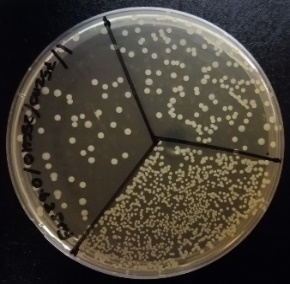  **(e)** | **(f)** | 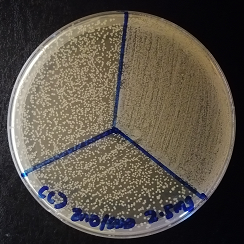  **(g)** |

**Figure S1.** MIC/MBC for BZCnanocomposites. **(a)** 10% DMSO as negative control, **(b)** B1Z3C at 5 mg/mL, **(c)** B1Z3C at 10 mg/mL, **(d)** B3Z1C at 2.5 mg/mL, **(e)** B3Z1C at 1.25 mg/mL, **(f)** B3Z1C at 0.625 mg/mL and **(g)** B3Z1C (commercial) at 2.5 mg/mL.

| **Pathogenic bacteria** | **B3Z1C**  **30 min** | **B3Z1C**  **1 h** | **B3Z1C**  **3 h** | **B3Z1C**  **6 h** | **B3Z1C**  **12 h** | **B3Z1C**  **24 h** |
| --- | --- | --- | --- | --- | --- | --- |
| **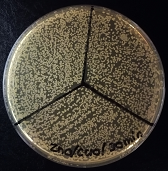**  ***S. aureus*** | **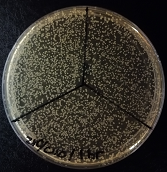**  **(a)** | **(b)** | 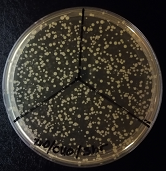  **(c)** | 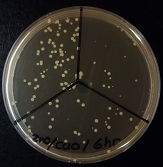  **(d)** | 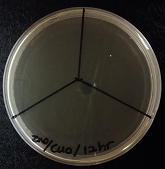  **(e)** | 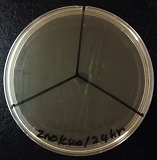  **(f)** |

**Figure S2.** Time-kill assay showing antimicrobial activity exhibited by 2.5 mg/mL of B3Z1C nanocomposites at different time against *S. aureus*. **(a)** 0.5 h (30 min), **(b)** 1 h, **(c)** 3 h, **(d)** 6 h, **(e)** 12 h and **(f)** 24 h.

| ***S. aureus*** | **Negative control** | **B1Z3C** | **B1Z1C** | **B3Z1C** | **B3Z1C (commercial)** | ***C*. *gigantea* extract** | **Positive control (antibiotics)** |
| --- | --- | --- | --- | --- | --- | --- | --- |
| **2.5 mg/mL** | 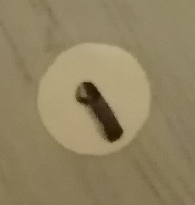 | 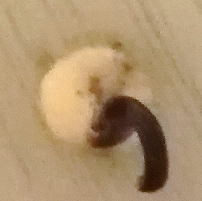 | 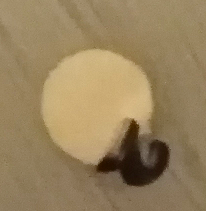 | 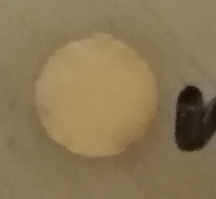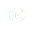  **(d)** | 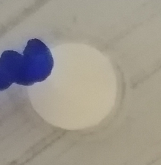  **(e)** | 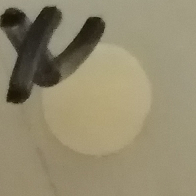  **(f)** | 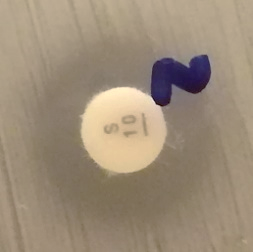  **(g)** |
| **10 mg/mL** | 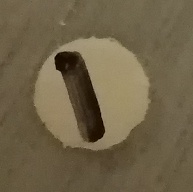  **(a)** | 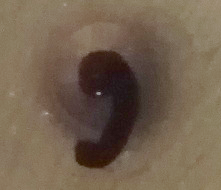  **(b)**  **(a)**  **(b)** | 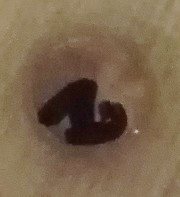  **(c)**  **(c)** | 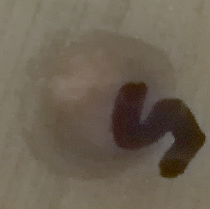  **(d)** | 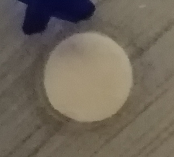  **(e)** | 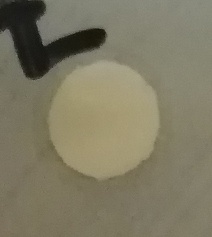  **(f)** |  |

**Figure S3.** Zone of inhibition (mm) exhibited by 2.5 mg/mL and 10 mg/mL of BZC nanocomposites against *S. aureus*. **(a)** Negative control, **(b)** B1Z3C, **(c)** B1Z1C, **(d)** B3Z1C, **(e)** B3Z1C (commercial), **(f)** *C*. *gigantea* extract and **(g)** Positive control (Streptomycin-10 µg). These data represent mean (± SD) of three replicates.
